# Metabolism of Natural Killer Cells and Other Innate Lymphoid Cells

**DOI:** 10.3389/fimmu.2020.01989

**Published:** 2020-08-28

**Authors:** Jingjing Cong

**Affiliations:** ^1^Hefei National Laboratory for Physical Sciences at Microscale, The CAS Key Laboratory of Innate Immunity and Chronic Disease, Division of Life Sciences and Medicine, School of Basic Medical Sciences, University of Science and Technology of China, Hefei, China; ^2^Institue of Immunology, University of Science and Technology of China, Hefei, China

**Keywords:** natural killer cells, innate lymphoid cells, metabolism, glycolysis, oxidative phosphorylation

## Abstract

Natural killer (NK) cells are the host's first line of defense against tumors and viral infections without prior sensitization. It is increasingly accepted that NK cells belong to the innate lymphoid cell (ILC) family. Other ILCs, comprising ILC1s, ILC2s, ILC3s and lymphoid tissue inducer (LTi) cells, are largely non-cytotoxic, tissue-resident cells, which function to protect local microenvironments against tissue insults and maintain homeostasis. Recently, evidence has accumulated that metabolism supports many aspects of the biology of NK cells and other ILCs, and that metabolic reprogramming regulates their development and function. Here, we outline the current understanding of ILC metabolism, and describe how metabolic processes are affected, and how metabolic defects are coupled to dysfunction of ILCs, in disease settings. Furthermore, we summarize the current and potential directions for immunotherapy involving targeting of ILC metabolism. Finally, we discuss the open questions in the rapidly expanding field of ILC metabolism.

## Introduction

Innate lymphoid cells (ILCs) represent a heterogeneous population of lymphocytes, which do not generate genetically rearranged antigen receptors, but instead express germline-encoded activating, and inhibitory receptors (1–4). Based on their requirements for developmental transcription factors, cell surface marker expression, and patterns of cytokine production, ILCs are classified into group 1 ILCs [including natural killer (NK) cells and ILC1s], group 2 ILCs (ILC2s), and group 3 ILCs [including ILC3s and lymphoid tissue inducer (LTi) cells] (1–5). ILCs regulate a wide variety of immunological processes. Specifically, NK cells are cytotoxic and defend against tumors and viral infections, mainly through production of cytotoxic granules, interferon (IFN)-γ, and tumor necrosis factor (TNF) (6). ILC1s are generally non-cytotoxic and respond to intracellular pathogens, such as viruses and *Toxoplasma gondii*, through secretion of IFN-γ and TNF (7–9). ILC2s produce the type 2 cytokines interleukin (IL)-4, IL-5, IL-13, and amphiregulin, which function in anti-helminth immunity, allergic inflammation, and tissue repair (10–14). ILC3s react to extracellular bacteria and fungi through the action of IL-17 and IL-22 (15–18), while LTi cells promote the development of secondary lymph nodes in a lymphotoxin-dependent manner (19). Further, recent evidence indicates that ILCs can mediate memory responses; a feature of adaptive lymphocytes (20–24).

**Figure 1:**
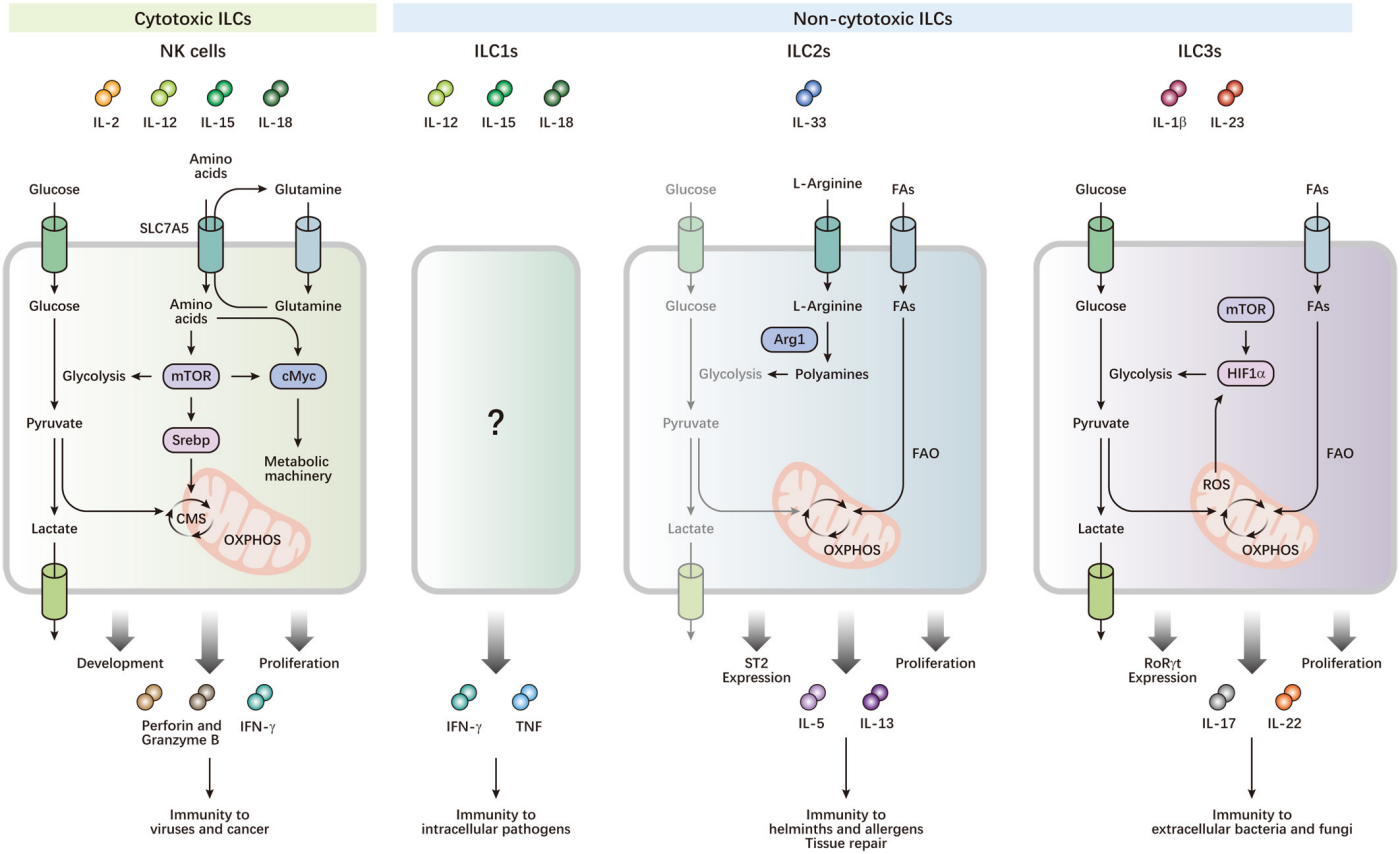
Metabolism drives the development and functions of ILCs. ILCs can be activated by cytokine signals from local microenvironments, and contribute to immune defense and maintenance of homeostasis. NK cells preferentially utilize glucose, which is metabolized by glycolysis and CMS, to fuel OXPHOS and ATP production, which power their effector functions and rapid proliferation. cMyc and Srebp are important metabolic regulators, which control the expression of metabolic machinery and CMS, respectively. mTOR activity is required for optimal Srebp activity and the initial induction of cMyc, while maintenance of cMyc protein levels depends on SLC7A5, which transports glutamine out of the cell, facilitating transport of other amino acids into the cell. Leucine imported by SLC7A5 improves mTOR activity. High mTOR activity is also critical for increased metabolism to support proliferative expansion during NK cell development. ILC2s preferentially generate energy by breaking down FAs, but not glucose, for OXPHOS to respond to helminths and allergens. Arg1 is required for ILC2 cytokine production and optimal proliferation through the conversion of L-arginine into polyamines. Arg1 also promotes ILC2 glycolytic capacity. The balance of metabolism between FAO and glycolysis is important for expression of the IL-33 receptor ST2, and production of IL-5 and IL-13 in ILC2s. Activated ILC3s exhibit increases in both glucose and FA metabolism. Rapid proliferation and effector functions of ILC3s require mTOR-HIF1α-dependent glycolysis and RORγt expression. Mitochondrial respiration and production of mitochondrial ROS stabilizes HIF1α, thereby consolidating ILC3 glycolysis.

Metabolic pathways are regulated by intrinsic and extrinsic signals to fulfill the energy and biosynthetic needs for cell growth, survival, and specialized functions (25). Glycolysis and mitochondrial oxidative phosphorylation (OXPHOS) are primary metabolic pathways for ATP generation (26). Glucose fuels both pathways and is first converted to pyruvate via glycolysis, then metabolized to lactate, or transported into the mitochondria for OXPHOS. Glutamine and fatty acids (FAs) can also fuel OXPHOS through glutaminolysis and fatty acid oxidation (FAO), respectively. Glycolysis generates two ATP molecules from one glucose molecule, whereas up to 36 ATP molecules are gained per single molecule of glucose via OXPHOS. Despite its low efficiency, glycolysis can rapidly produce energy and also supplies biosynthetic precursors that facilitate the survival of rapidly proliferating cells (27, 28). In the context of immunity, appropriate metabolic alterations are required in immune cells to ensure a productive immune response; for example, production of cytotoxic granules and cytokines by NK cells and non-NK ILCs (25, 29–33). In contrast, dysregulation of NK cell metabolism leads to their dysfunction, and is often associated with disease (34–37). In this review, we provide an overview of the current understanding and gaps in knowledge regarding the metabolism of NK cells and other ILCs (Figure 1), and consider novel therapeutic strategies in which ILC metabolism could be targeted.

## Basic Nk Cell Metabolism

At steady state, murine NK cells display a metabolically quiescent phenotype and preferentially utilize glucose-fueled OXPHOS (38, 39). This inactive but efficient metabolic pattern is sufficient to meet their biosynthetic and energy demands, even in the context of activation by short-term cytokine stimulation or activating receptor ligation (39). Low levels of glycolysis and OXPHOS are also observed in unstimulated human peripheral blood NK cells (40).

In humans, NK cells are generally divided into two subsets, according to expression of CD56: CD56^dim^ cells and CD56^bright^ cells. The vast majority of NK cells in the peripheral blood are CD56^dim^ NK cells, which have a well-differentiated phenotype and potent cytotoxicity, whereas the less mature CD56^bright^ NK cells are predominant in secondary lymphoid tissues and are the main producers of cytokines (41–44). Recent studies have shown that the two subsets are metabolically distinct, which, at least in part, accounts for their different functions. In response to cytokine stimulation, CD56^bright^ NK cells, probably by virtue of higher cytokine receptor expression, show more pronounced metabolic changes and are more sensitive to metabolic inhibition than CD56^dim^ NK cells (40). The CD56^bright^ NK cells can be further divided into tissue-resident and circulating subsets. Liver- and spleen-resident CD56^bright^ subsets exhibit lower expression of the glucose transporter GLUT1, but higher expression of the L-amino acid transporter CD98, following IL-12/IL-15 stimulation, relative to CD56^bright^ NK cells from paired peripheral blood (45). These differential expression profiles of nutrient transporters in NK cells from different tissues may relate to the preference of NK cells for specific nutrients in these compartments, which may further influence the circulating and tissue-resident characteristics of NK cells. Identifying the unique metabolic profile of NK cells in diverse tissues will help to solve this issue.

Mouse NK cells mainly originate and develop in the bone marrow, and then migrate to peripheral tissues. They are characterized into four subsets, based on the expression of CD27 and CD11b, from immature to mature: CD27^−^CD11b^−^, CD27^+^CD11b^−^, CD27^+^CD11b^+^, and CD27^−^CD11b^+^ (46– 49). The metabolic activity of NK cells changes dynamically during development (38). Immature NK cells have higher metabolic activity, which is important to support their proliferative expansion. As NK cells mature, pathways related to proliferation are downregulated, and there is a concomitant decrease in the expression of CD98 and the transferrin receptor CD71, as well as glucose uptake, while pathways associated with quiescence are upregulated (38).

Further, during NK cell maturation, they undergo an “education” process, which is generally mediated by inhibitory receptors (KIR in humans and Ly49 in mice), and confers NK cell functional competence, as well as their capacity to distinguish between normal and abnormal cells (50, 51). In humans, metabolic reprogramming toward glycolysis and OXPHOS supports the cytotoxic responses of educated NK cells, while uneducated NK cells rely entirely on OXPHOS for this function (52). Thus, activation of glycolysis may be an important mechanism that accounts for the functional difference between educated and uneducated NK cells. In mice, Marcais et al. found that mechanistic target of rapamycin (mTOR) activity is essential for NK cell education, and that the higher mTOR activity in educated NK cells contributes to maintenance of their optimal reactivity and metabolic parameters (53).

Metabolic regulation in NK cells is driven by intricate molecular mechanisms, among which mTOR is the most widely studied. mTOR is a serine threonine kinase and functions as two different complexes, mTORC1 and mTORC2, that differ in their regulation and targets (54). mTORC1 activity is important for the development and functions of NK cells through regulating metabolism; however, little is known about mTORC2 in this regard. In mice, mTOR activity is higher in CD27^+^CD11b^−^ NK cells than CD27^+^CD11b^+^ and CD27^−^CD11b^+^ subsets, suggesting a progressive loss of mTOR activity as NK cells mature (38). Decreased mTOR activity of mature NK cells is associated with their reduced metabolism and proliferative potential (38, 49). Indeed, mTOR-deficient NK cells exhibit a drastic block of differentiation in the bone marrow at the CD27^+^CD11b^−^ to CD27^+^CD11b^+^ stage, due to compromised proliferation (38).

mTOR activity is required for increased glycolysis and mitochondrial functions during NK cell activation. Pharmacological inhibition of mTOR by rapamycin reduces the upregulation of glycolysis in mouse NK cells stimulated by IL-2/IL-12, and leads to a decrease in mitochondrial mass and membrane potential in human NK cells stimulated by IL-2, resulting in impaired effector functions (36, 55). Consistently, CD56^bright^ NK cells that exhibit stronger metabolic responses to IL-2 or IL-12/IL-15 stimulation, have higher mTOR activity compared with CD56^dim^ NK cells (40); however, excessive activation of mTOR can cause mitochondrial fragmentation, thereby damaging mitochondrial function (37).

mTOR is also involved in metabolic regulation via other molecules, such as sterol regulatory element-binding protein (Srebp) and cMyc. Srebp and cMyc control activation-induced metabolic reprogramming in NK cells, and mTOR is required for optimal Srebp activity and the initial induction of cMyc (56, 57).

## Metabolism And Nk Cell Function

It is increasingly accepted that cellular metabolism is indispensable for the activation and specialized functions of immune cells (58, 59). For NK cells, metabolic reprogramming is closely involved in multiple immunological processes, where metabolic changes facilitate the generation of energy and biosynthetic precursors to achieve specialized functions.

### Effector Function

NK cells are the first line of defense against infected and transformed cells by production of cytokines and release of cytotoxic granules. NK cell effector functions are controlled by the cytokine milieu, and integration of activating and inhibitory signals on targets (6, 60). Acquisition of full effector functions in NK cells requires substantial metabolic reprogramming. Different activation stimuli cause distinct cellular metabolic changes. Generally, NK cell activation by receptor ligation is more metabolically dependent than that in response to cytokine stimulation (39).

In the case of IL-2/IL-12 activation, activated mouse NK cells exhibit increased glycolysis, OXPHOS, glucose uptake, and lipid synthesis (55, 56). Glucose is the primary fuel driving enhanced glycolysis and OXPHOS in such activated NK cells. Following cytokine stimulation, glucose is first converted to pyruvate through glycolysis, and then some pyruvate is metabolized to lactate; however, unlike in other lymphocytes, almost no pyruvate is fed into the tricarboxylic acid (TCA) cycle. In NK cells, this pyruvate is metabolized to cytosolic citrate via the citrate-malate shuttle (CMS) to fuel OXPHOS and ATP synthesis, a process controlled by Srebp (56). Inhibition of glycolysis or CMS significantly diminishes IFN-γ production and Granzyme B expression in NK cells (55, 56). Although lipid synthesis is essential for the metabolic reprogramming of effector T cells, inhibition of lipid-synthesis pathways has minimal effects on NK cell effector functions and proliferation (56, 61, 62). Glutamine is another important fuel for metabolically active cells, as glutaminolysis feeds into the TCA cycle, and is not a critical fuel for powering OXPHOS in activated NK cells. Nevertheless, glutamine availability is essential for NK cell metabolism and effector functions as it contributes to sustaining cMyc levels (57). cMyc plays an important role in the metabolic regulation of NK cells because it controls the expression of metabolic machinery, including glucose transporters and glycolytic enzymes, which are required to support increased metabolism during NK cell activation (57). Glutamine helps the uptake of amino acids essential for high rates of cMyc protein synthesis through the L-type amino acid transporter SLC7A5, counteracting continuous cMyc degradation, and thereby maintaining cMyc expression in NK cells. Withdrawal of glutamine or blockade of amino acid transport, leads to cMyc loss and concomitant impaired effector functions (57). As important nutrients, whether FAs can fuel NK cells remains unknown. In fact, FA administration can suppress NK cell effector functions and metabolism (35). Thus, NK cells preferentially utilize glucose, which is metabolized by glycolysis and CMS, to power effector functions. Mechanistically, metabolic activation is essential for NK cells to produce effector protein, including IFN-γ and Granzyme B, and to form a correct immunological synapse with target cell (35, 55).

In the case of IL-2 or IL-12/IL-15 activation, CD56^bright^ NK cells preferentially upregulate the nutrient receptor CD71 and CD98 in an mTOR-dependent manner, compared with CD56^dim^ NK cells (40). Further, CD56^bright^ NK cells express higher levels of GLUT1 at baseline, allowing them to rapidly uptake glucose upon activation. Inhibition of glycolysis significantly diminishes IFN-γ production by CD56^bright^ NK cells, while it had minimal effect on degranulation or Granzyme B induction in CD56^bright^ or CD56^dim^ NK cells (40). Overall, CD56^bright^ NK cells are more metabolically active than their CD56^dim^ NK counterparts, and this assists CD56^bright^ NK cells in their rapid production of large amounts of IFN-γ during immune responses.

The effect of IL-15 on NK cell function is dose-dependent; at low concentrations it maintains their survival, while at higher concentrations it promotes activation and proliferation (63–65). Accordingly, a dose-dependent effect of IL-15 on NK cell metabolism is also observed. Recent studies have found that low doses of IL-15 do not induce metabolic changes, while high doses of IL-15 increase glycolysis and OXPHOS in NK cells, with a bias toward glycolysis, possibly because only high doses of IL-15 trigger mTOR signaling (38, 39). Interestingly, continuous exposure to low doses of IL-15 exhausts NK cells via repressing their metabolism (66). In terms of *in vivo* functions, glycolysis is essential for NK cell cytotoxicity and murine cytomegalovirus (MCMV) control, but it does not influence IFN-γ production, which suggests that immunometabolism has different effects on NK cell cytotoxicity and cytokine secretion (67). Moreover, IL-15 priming can reduce this glycolytic requirement for NK cell cytotoxicity, highlighting the therapeutic potential of IL-15 in viral infections (67).

### Memory Formation

Immune memory has long been considered a characteristic of the adaptive immune system; however, recent studies have demonstrated that NK cells also generate long-term memory responses against acute viruses, haptens, and cytokine stimulation (20–22). After exposure to stimuli, NK cells undergo expansion and contraction, and eventually form a pool of memory NK cells, with enhanced function, upon encountering the same stimuli. Using a mouse model of MCMV infection, O'Sullivan et al. found that mitochondrial quality in NK cells exhibited dynamic changes from the clonal expansion phase to the memory phase (68). The proliferative burst of NK cells leads to mitochondrial depolarization and accumulation of mitochondrial-associated reactive oxygen species (ROS). During the subsequent contraction-to-memory phase transition, a protective autophagic process, called mitophagy, is induced, which promotes the generation of NK cell memory through removal of dysfunctional mitochondria and ROS (68). Inhibition of mTOR by rapamycin or activation of AMPK by metformin increases autophagic activity, and this further improves the survival of memory NK cells (68). Similarly, metformin also facilitates memory formation in mouse CD8^+^ T cells (69). There is evidence that mitochondrial FAO is essential for memory CD8^+^ T cell development, and that metformin stimulates FAO in CD8^+^ T cells during viral infection (69, 70). Furthermore, autophagy deficiency in CD8^+^ T cells leads to dysregulated mitochondrial FAO (71). Thus, it will be interesting to investigate the relationship between FAO, mitophagy, and NK cell memory.

There have been recent reports that NKG2C^+^ NK cells, which highly co-express CD57, expand and persist in the peripheral blood of humans infected with human cytomegalovirus (HCMV). These cells possess memory-like properties, and are referred to as adaptive NK cells (72–74). Compared with non-adaptive NK cells, adaptive NK cells display a more metabolically active phenotype, mainly manifested as increased glycolysis, mitochondrial respiration and mitochondrial membrane potential, elevated ATP synthesis, and increased glucose uptake (75). Mechanistically, adaptive NK cells upregulate the expression of chromatin-modifying transcriptional regulator AT-rich interaction domain 5B (ARID5B), which enhances mitochondrial metabolism by inducing genes encoding components of the electron transport chain, highlighting a link between epigenetics and metabolism (75).

In other studies, it has been demonstrated that NK cells that recall respiratory influenza virus and skin contact hypersensitive chemical hapten reside in the liver, but not in the infection or sensitization site (20, 76). Wang et al. further demonstrated that hapten-specific memory NK cells are generated in the lymph nodes (23, 77). These findings raise the question of whether the formation and long-term maintenance of memory NK cells requires a unique nutritional and metabolic environment, which differs among tissues. Furthermore, it remains unclear whether there are variations in the metabolism of memory NK cells induced by different stimuli, such as cytokines and haptens.

## Nk Cell Metabolism in Disease

NK cell function and metabolism are highly integrated. Dysregulated cellular metabolism of NK cells has been documented in cancer, obesity, and chronic viral infection, and is an important cause of NK cell dysfunction in these diseases.

### Obesity

Obesity is associated with an increased incidence of cancer and infections (78–80), which may, at least in part, be due to NK cell dysfunction, since NK cells in the peripheral blood of obese humans (both adults and children) exhibit reduced cell frequencies, diminished cytotoxicity, and impaired IFN-γ production (35, 81, 82). Similarly, downregulated effector molecule expression was observed in spleen NK cells from obese mice fed on high-fat diet (HFD) (35). One recent study illustrated how obesity affects NK cell function by regulating intrinsic cellular metabolism (35). Obesity induces robust activation of peroxisome proliferator-activated receptor (PPAR), which contributes to NK cell uptake of lipids. This lipid accumulation inhibits the mTOR pathway, cMyc expression, and activation-induced metabolic reprogramming, leading to loss of NK cell function (35).

Unlike spleen and peripheral blood, adipose NK cells are overactivated in obese mice fed on HFD. These NK cells expand faster and produce more IFN-γ and TNF, which induces the formation of proinflammatory macrophages and further promotes insulin resistance and inflammation (83, 84). This seeming paradoxical state may be caused by their unique local microenvironments, as obesity increases the expression of the ligands of NK cell activating receptor NCR1 on adipocytes, thereby stimulating the IFN-γ production and local expansion of NK cells (83). Moreover, obesity could drive the secretion of IL-15 by adipose tissue macrophages, which has been demonstrated to have a positive effect on NK cell metabolism, proliferation and activation (84). In addition to NK cells, it is recently found that IL-12 activation of ILC1s is also implicated in driving obesity-associated adipose tissue inflammation (85, 86). In summary, these studies suggest that obesity leads to the overaction of adipose NK cells, but loss of function of peripheral NK cells, which further contributes to obesity-related pathology and attenuated immunosurveillance, respectively.

### Cancer

**Figure 2:**
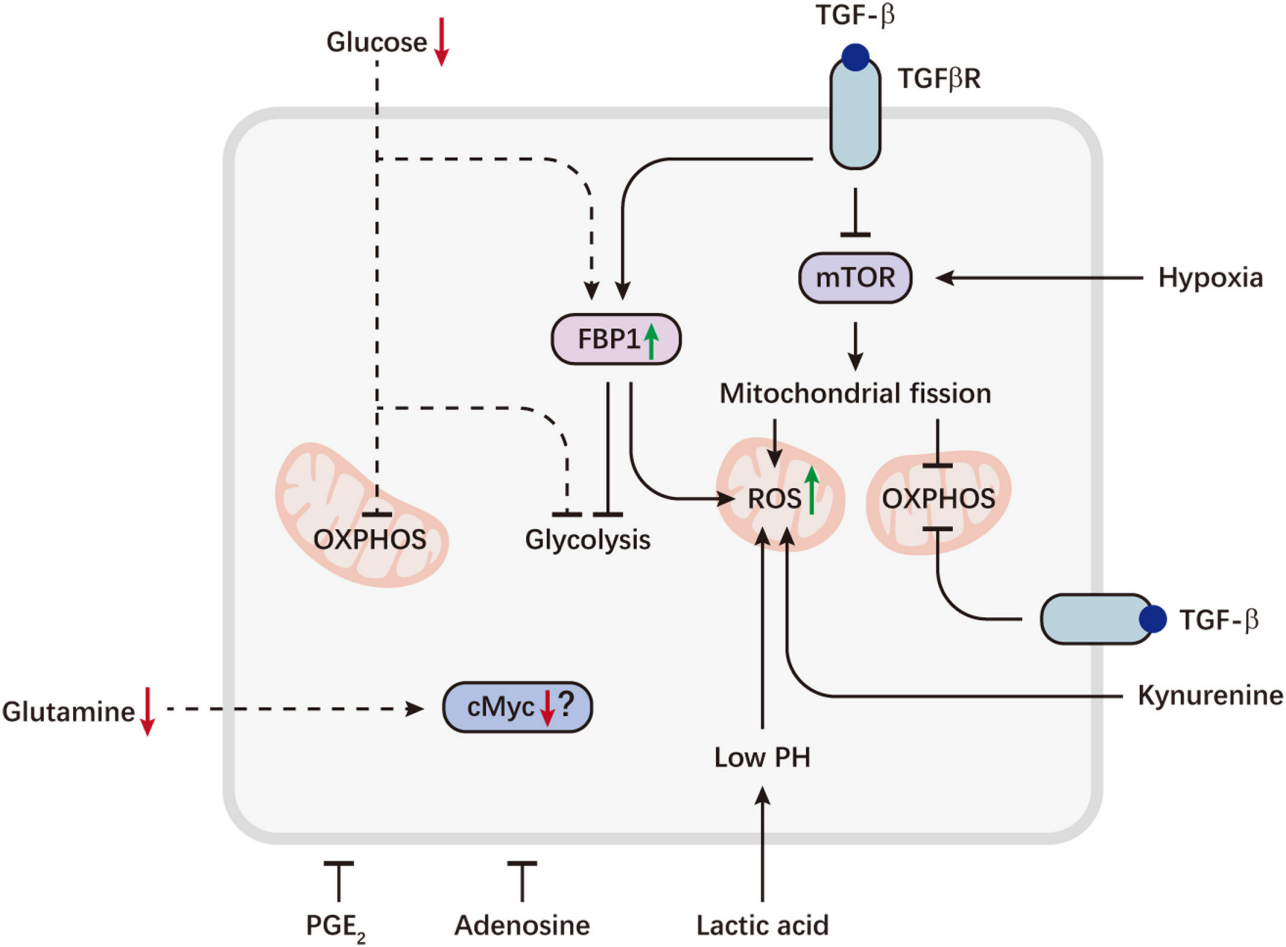
Metabolic reprogramming of NK cells in the tumor microenvironment. Tumor cells can evade immunosurveillance by repressing NK cell metabolism. Aberrant expression of the gluconeogenic enzyme FBP1, caused by TGFβ, can inhibit NK cell glycolysis and promote ROS production. TGFβ also directly inhibits mTOR and drives mitochondrial dysfunction. On the other hand, sustained activation of mTOR induced by hypoxia, can lead to excessive fragmentation of mitochondria via fission, which is associated with decreased OXPHOS and increased ROS levels. Tumor-imposed metabolic restrictions may be involved in NK cell dysfunction. Depletion of glucose, a key fuel for NK cells, may induce FBP1 expression or directly restrict glucose utilization by NK cells, resulting in defective glycolysis and OXPHOS. Decreased availability of glutamine may lead to a loss of cMyc protein, thereby inhibiting NK cell metabolism. Immunosuppressive metabolites accumulated in the tumor microenvironment, including lactic acid, kynurenine, PGE_2_ and adenosine, are potent inhibitors of NK cell viability and effector functions. Lactic acid causes decreased ATP synthesis and intracellular acidification, resulting in accumulation of ROS. Kynurenine can also induce NK cell apoptosis via ROS. Dashed lines indicate likely (unproven) interactions.

NK cells provide potent protection from tumors; however, tumor cells can evade immunosurveillance by inducing NK cell dysfunction through a range of mechanisms. Indeed, NK cells isolated from tumors are decreased in cell number and/or exhibit impaired functions compared with non-tumor NK cells in patients with lung cancer, liver cancer, prostate cancer, pancreatic cancer, and colorectal liver metastasis (37, 87–91). TGF-β is a pleiotropic cytokine that is frequently upregulated in tumors, and is believed to have a negative impact on NK cell function. Inhibition of cellular metabolism is an important mechanism responsible for TGF-β-induced NK cell dysfunction in the tumor microenvironment (Figure 2) (92). Our previous findings showed that the gluconeogenic enzyme fructose-1,6-bisphosphatase (FBP1) is induced in NK cells during tumor development in a *Kras*-driven lung cancer model. Aberrant FBP1 expression, potentially caused by TGF-β, elicited NK cell dysfunction by suppressing glycolysis and compromising cell viability. Notably, pharmacological inhibition of FBP1 could restore glycolysis, and thus improve NK cell effector functions (34). On the other hand, Slattery et al. found that peripheral blood NK cells from patients with metastatic breast cancer had impaired mTOR activity, profound metabolic defects, and altered mitochondrial morphology. Blocking elevated TGF-β *ex vivo* improved mTOR activity, metabolic parameters, and IFN-γ production of NK cells in these patients (36).

Rapid proliferation is a hallmark of cancer. The rapid proliferation of tumor cells leads to a lack of glucose, glutamine, and oxygen, while there is accumulation of plentiful metabolites, such as lactic acid, in the tumor microenvironment (93–97). Recent work from Zheng et al. underlined a detrimental role of hypoxia in NK cell mitochondrial metabolism. They found that tumor NK cells had small, fragmented mitochondria in patients with liver cancer, which was a consequence of hypoxia-induced excessive mitochondrial fission. This mitochondrial fragmentation impaired the cytotoxicity and survival of NK cells, thereby resulting in immune evasion (37).

Glucose consumption by tumor cells weakens T cell glycolysis and their capacity to produce IFN-γ in a mouse sarcoma model (94). Although NK cells generate energy primarily by breaking down glucose to fuel glycolysis and OXPHOS (39, 56), it remains unclear how tumor-mediated glucose restriction impacts NK cell metabolism and function. As FBP1 is a critical enzyme for gluconeogenesis, a process that generates glucose, it is reasonable to predict that aberrant FBP1 expression in NK cells may also be a consequence of glucose deprivation. In addition, given the importance of glutamine availability for sustaining cMyc expression (57), tumor-driven glutamine deprivation may dampen NK cell antitumor function by inhibiting metabolism. However, it is necessary to investigate the authentic effects of tumor-imposed metabolic restrictions on NK cells to validate these hypotheses and guide development of effective treatment.

Lactic acid accumulated in the tumor microenvironment is a potent inhibitor of NK cell effector function and viability (90, 98–100). Intracellular acidification and decreased ATP synthesis caused by lactic acid may be related to impaired IFN-γ production by NK cells (99). A more recent study in patients with colorectal liver metastasis showed that tumor-derived lactic acid led to accumulation of mitochondrial ROS by decreasing intracellular pH, leading to apoptosis of liver-resident NK cells (90). Other metabolites, such as adenosine, prostaglandin E_2_ (PGE_2_), and kynurenine, which are frequently increased in the tumor microenvironment, also repress NK cell function, and kynurenine has been reported to cause human NK cell apoptosis via ROS (101–105).

### Chronic Viral Infection

In the context of chronic infections, such as with human immunodeficiency virus 1 (HIV-1) and hepatitis B virus (HBV), the immune system fails to clear the virus, leaving NK cells exposed to persistent inflammation. This scenario results in deterioration of NK cell effector functions and is often accompanied by upregulation of inhibitory receptors, such as PD-1, NKG2A, and Tim-3, a status akin to CD8^+^ T cell exhaustion (106–109). Further, a recent study by Cubero et al. showed that, during chronic HIV-1 infection, NK cells exhibited metabolic defects bearing similarities to exhausted CD8^+^ T cells (110). Persistent HIV-1 infection led to NK cell mitochondrial dysfunction, characterized by defective OXPHOS, increased mitochondrial depolarization and fragmented mitochondria, which limited the ability of NK cells to mobilize alternative fuels to power IFN-γ production upon activating receptor stimulation; a state of metabolic inflexibility. IL-15 pre-treatment could bypass the metabolic requirement of NK cells for receptor ligation and potentiate NK cell functions. Indeed, activation of NK cells by stimulation of activating receptors has been demonstrated to be an energy-demanding process (39, 110).

Recent research has linked the increased expression of inhibitory receptors to compromised cellular metabolism. For example, PD-1 represses glycolytic and mitochondrial metabolism via inhibiting the expression of a critical metabolic regulator, thereby exhausting CD8^+^ T cells (111). Another study identified a role of LAG-3 in limiting CD4^+^ T cell mitochondrial biogenesis (112). As multiple inhibitory receptors, including PD-1, are upregulated on NK cells during chronic viral infection, they may also negatively regulate the metabolic fitness of NK cells.

## Metabolism Of Other Ilcs

Like Th1 cells, ILC1s are non-cytotoxic innate helper lymphoid cells, which defend against intracellular pathogens through production of IFN-γ and TNF. There is evidence that NK cells can be converted into ILC1s under pathological conditions, such as tumor growth, and TGF-β is a critical driver (113, 114). The fact that TGF-β has been demonstrated to regulate cellular metabolism suggests that metabolic reprogramming may be implicated in controlling the plasticity of group 1 ILCs (36, 115, 116). Indeed, direct killing of abnormal target cells is an energy-demanding process, and ILC1s hardly possess such capacity (35, 114, 117). In contrast, ILC1s in the tumor microenvironment exhibit increased TNF production, which is independent of mTOR and glycolytic pathways (55, 114). Furthermore, compared with tumor NK cells, tumor ILC1s express higher levels of the genes encoding PD-1 and LAG-3, which repress bioenergetics and mitochondrial biogenesis in T cells (111, 112, 114). These findings indicate that low metabolic activity may be sufficient to support the requirements for ILC1 survival and function, and this confers them a survival advantage in the nutrient-deprived tumor microenvironment. However, in most cases, such as viral infection and inflammation, NK cells are not converted to ILC1s (9, 118–121), and the metabolic pathways used by such ILC1s to response to pathogens remain to be determined.

It is widely accepted that glycolysis is indispensable to fuel NK cell proliferation and effector functions, and emerging evidence suggests that glycolysis is also closely involved in the regulation ILC2s and ILC3s. Monticelli et al. found that ILC2s showed elevated glycolytic capacity in comparison to Th2 cells in response to IL-33, indicating that activated ILC2s have the potential to engage glycolysis (122); however, a shift in the balance of metabolism from OXPHOS toward increased glycolysis led to defective in ILC2 maturation and function (123, 124). These results imply that glycolysis may act as a rheostat that modulates ILC2 immune responses. Transcriptional analysis demonstrated that resting ILC3s are enriched for pathways associated with carbohydrate metabolism and glycolysis, consistent with the finding that mTOR is essential for the generation of ILC3s (38, 125). Moreover, ILC3s rely on mTOR-HIF1α axis-induced glycolysis, as well as mitochondrial ROS, for proliferation and cytokine secretion after *in vitro* activation by IL-1β and IL-23 or during bacterial infection (126). Nevertheless, HIF1α is not required to support increased glycolysis in activated NK cells (57). In addition to glucose metabolism, FA metabolism also plays an important role in ILC3 function. FAO contributes to the production of IL-17 and IL-22 by ILC3s, and elevated FA uptake has been observed in activated ILC3s (126). FA synthesis is dispensable for survival and proliferation of RORγt^+^ ILCs (ILC3s), but it promotes RORγt^+^ ILC-derived IL-22 expression during intestinal infection. Blockade of FA synthesis by targeting the key enzyme acetyl-CoA carboxylase 1 (ACC1) in these cells can dampen epithelial defense mechanisms by interfering with IL-22 production (127). In line with these findings, Di Luccia et al. also demostrated that FA synthesis is evident in activated ILC3s, which is a metabolic feature different from Th17 cells (126).

Recent studies have demonstrated the critical role of amino acid and FA metabolism in ILC2-mediated type 2 immune responses (122, 128). The metabolic enzyme arginase-1 (Arg1), which metabolizes L-arginine into urea and ornithine to yield a range of downstream metabolites critical for cellular and bioenergetic processes, is constitutively expressed in precursor and mature ILC2s (122). Deletion of ILC-intrinsic, but not myeloid cell-intrinsic, Arg1 restrains ILC2 responses and dampens type 2 inflammation in the lung (122). Mechanistically, Arg1 is required for optimal ILC2 proliferation and cytokine secretion, and inhibition of Arg1 prevents conversion of L-arginine into polyamines, which can regulate cell growth and survival (122). Arg1 promotes the glycolytic capacity of activated ILC2s, but minimally influences their OXPHOS, indicating a role for Arg1 in the regulation of glucose metabolism. Although Arg1 is also expressed in a proportion of ILC3s, it is not required for ILC3 development and function (122).

Another study identified genes involved in FA metabolism as a metabolic feature of intestinal ILC2s and, accordingly, intestinal ILC2s constitutively acquire large amounts of extracellular FAs (128, 129). FAO is dispensable for ILC2 homeostasis; however, abrogation of FAO significantly impairs intestinal ILC2 accumulation and production of IL-5 and IL-13 during helminth infection (128). Nevertheless, blocking glucose utilization does not impact ILC2 accumulation and function, suggesting that ILC2s mainly generate energy by breaking down FAs for OXPHOS, to protect against helminth infection (128). In addition to intestinal ILC2s, FAO is also important for mediation of allergic inflammation by lung ILC2s (124). In the absence of vitamin A or its metabolite retinoic acid, there is an increase in the number and function of ILC2s, caused by their elevated FA uptake and enhanced FAO (128, 130). In contrast, vitamin A and retinoic acid promote intestinal ILC3 accumulation and function (130, 131). These findings are interesting, as lipids and vitamin A have different effects on NK cells, ILC2s, and ILC3s, suggesting that the distinct metabolic profiles of each type of ILC are coupled to their unique characteristics.

## Targeting Ilc Metabolism For Therapy

Dysfunction of ILCs can lead to defective immunosurveillance or excessive inflammation, which are important causes of many diseases, and adjusting ILC function through manipulation of metabolism is a potential direction for therapy. There are several potential ways in which ILC metabolism could be targeted.

### Cell-Intrinsic Metabolic Regulators and Pathways

As mentioned earlier, NK cell and non-NK ILC functions are controlled by a range of metabolic regulators and pathways. Inhibiting PPAR or blocking lipid transport into mitochondria restores NK cell cytotoxicity during obesity, which might improve cancer outcomes in such patients (35). Tumor-induced FBP1 and mitochondrial fragmentation account for impaired NK cell anti-tumor functions by compromising glycolysis and mitochondrial respiration, respectively, and targeting them can enhance NK cell-based tumor immunosurveillance (34, 37). Dysregulation of mTOR activity is observed in diverse tumors. mTOR signaling is excessive activated in tumor NK cells from patients with liver cancer, but is inhibited in peripheral blood NK cells from patients with metastatic breast cancer, and such aberrant mTOR activity is a critical driver of NK cell dysfunction (36, 37). Thus, mTOR activity modulation is another strategy that helps NK cells to achieve optimal function. As cMyc controls the expression of the metabolic machinery required for NK cell effector functions, therapeutic strategies that sustain cMyc protein levels, through inhibiting its degradation [by glycogen synthase kinase 3 (GSK3) blockade] or increasing glutamine availability (by glutaminase blockade), may contribute to improved immunosurveillance (57). Indeed, GSK3-inhibited NK cells show increased efficacy in multiple tumor models, although it remains unclear whether a metabolism-related mechanism is involved (132, 133). For ILC2-mediated type 2 immune inflammation, modulation of Arg1 enzymatic activity and fuel dependency are potentially new therapeutic approaches.

### Cytokines and Metabolites

TGF-β represses NK cells through multiple mechanisms, suggesting that blocking the interaction between NK cells and TGF-β could be a promising approach for protecting the metabolism and functions of NK cells in TGF-β rich environments, such as cancers. Recent studies found that NK cells genetically modified with a dominant-negative TGF-β receptor II resisted the suppressive effect of TGF-β and retained their potent ability to kill glioblastoma and breast tumor cells (134, 135). Such modified NK cells may also have improved metabolic parameters, as another study showed that blocking TGF-β *ex vivo* using neutralizing antibody restored the effector function and mitochondrial metabolism of NK cells from patients with metastatic breast cancer (36). In addition to TGF-β, there are soluble immunosuppressive metabolites, such as lactic acid, kynurenine and PGE_2_, in tumor microenvironments; therapeutic strategies that target these metabolites, through inhibition of lactate dehydrogenase, indoleamine 2,3-dioxygenases, and cyclooxygenase 2, respectively, may invigorate NK cell-based tumor immunosurveillance (99, 136, 137).

On the other hand, IL-15 priming has been demonstrated to reduce NK cell reliance on cellular metabolism, hence, IL-15 agonists may be effective for enhancing NK cell function in metabolically challenging environments, including viral infections and cancers (67, 110).

### Immune Checkpoints

Emerging immune checkpoint blockade strategies are being tested for their potential to reverse NK cell dysfunction in cancer and chronic viral infections, and there is evidence that anti-PD-1 or anti-PD-L1 antibody can enhance the anti-tumor efficacy of NK cells in multiple tumor models (138, 139). Given that the positive effects of PD-1/PD-L1 blockade on T cells are partly attributable to restoration of glycolytic and mitochondrial metabolism (111), this metabolism-related mechanism may also be involved in the reversal of NK cell dysfunction by checkpoint blockade.

## Concluding Remarks

Although the importance of cellular metabolism for immune function has been well-documented, the study of ILC metabolism is newly emerging. Over the past decade, evidence has mounted that cellular metabolism supports many aspects of the biology of NK cells and other ILCs, and that metabolic changes can dominate their immune responses; however, several major issues remain to be solved, including: (i) ILCs are the innate counterparts of T cells, and ILC1s mirror Th1 cells. Given the importance of metabolism in the survival and functions of T cells including Th1 cells, it will be interesting to investigate the major metabolic pathways utilized by ILC1s as well as LTi cells in the context of homeostasis and disorders. (ii) ILC plasticity has been demonstrated to be essential to shape ILC responses to diverse stimuli (113, 114, 140), which confers ILC subsets the capacities to change their phenotype and functions under specific circumstances, but nothing is known about the authentic metabolic alterations required for ILC plasticity. (iii) Recent findings show that functions of NK cells are markedly influenced by their local microenvironments, and that the unique characteristics of NK cells in different tissues have been uncovered step by step (141, 142). Other ILCs are largely tissue-resident cells, and this tissue dwelling property and the specialized distribution of non-NK ILCs are coupled to their functional heterogeneity (1). However, what are the differences in the metabolism of ILCs from diverse tissues, and how ILC metabolism is shaped by tissue microenvironments remains unclear at present. (iv) Whether and how intrinsic metabolism controls the circulating and tissue-resident characteristics of ILCs? (v) In addition to immune defense, NK cells can mediate immune tolerance and immune regulation; for example, promoting positive pregnancy outcomes through the secretion of growth-promoting factors, angiogenesis-regulating molecules, and cytokines, or maintaining liver immunotolerance through the regulation of dendritic cell-mediated tolerogenic regulatory T cell induction (143–147). However, how metabolic regulation is involved in immune tolerance and immune regulation mediated by NK cells, and the role of metabolism in controlling NK cell function switch, remains to be determined. (vi) How NK cell metabolism is involved in other diseases, in which NK cell abnormalities have been demonstrated, such as allergy and autoinflammatory diseases? A more thorough understanding of ILC metabolism will provide deep insights into ILC biology and aid the development of effective therapeutic strategiesbased on ILCs.

## Author Contributions

JC designed the review, wrote, and revised the manuscript.

## Conflict of Interest

The author declares that the research was conducted in the absence of any commercial or financial relationships that could be construed as a potential conflict of interest.
